# Tpl2 Protects Against Fulminant Hepatitis Through Mobilization of Myeloid-Derived Suppressor Cells

**DOI:** 10.3389/fimmu.2019.01980

**Published:** 2019-08-20

**Authors:** Jing Xu, Siyu Pei, Yan Wang, Junli Liu, Youcun Qian, Mingzhu Huang, Yanyun Zhang, Yichuan Xiao

**Affiliations:** ^1^The First Affiliated Hospital of Soochow University, Institutes for Translational Medicine, State Key Laboratory of Radiation Medicine and Protection, Key Laboratory of Stem Cells and Medical Biomaterials of Jiangsu Province, Medical College of Soochow University, Soochow University, Suzhou, China; ^2^CAS Key Laboratory of Tissue Microenvironment and Tumor, Shanghai Institute of Nutrition and Health, Shanghai Institutes for Biological Sciences, University of Chinese Academy of Sciences, Chinese Academy of Sciences, Shanghai, China; ^3^Department of Medical Oncology, Fudan University Shanghai Cancer Center, Shanghai, China

**Keywords:** hepatitis, myeloid-derive suppressor cells (MDSCs), TPL2, IL-25, chemokine

## Abstract

Myeloid derived suppressor cells (MDSC) in the liver microenvironment protects against the inflammation-induced liver injury in fulminant hepatitis (FH). However, the molecular mechanism through which MDSC is recruited into the inflamed liver remain elusive. Here we identified a protein kinase Tpl2 as a critical mediator of MDSC recruitment into liver during the pathogenesis of *Propionibacterium acnes*/LPS-induced FH. Loss of Tpl2 dramatically suppressed MDSC mobilization into liver, leading to exaggerated local inflammation and increased FH-induced mortality. Mechanistically, although the protective effect of Tpl2 for FH-induced mortality was dependent on the presence of MDSC, Tpl2 neither directly targeted myeloid cells nor T cells to regulate FH pathogenesis, but functioned in hepatocytes to mediate the induction of MDSC-attracting chemokine CXCL1 and CXCL2 through modulating IL-25 (also known as IL-17E) signaling. As a consequence, increased MDSC in the inflamed liver specifically restrained the local proliferation of infiltrated pathogenic CD4^+^ T cells, and thus protected against the inflammation-induced acute liver failure. Together, our findings established Tpl2 as a critical mediator of MDSC recruitment and highlighted the therapeutic potential of Tpl2 for the treatment of FH.

## Introduction

Fulminant hepatitis (FH) is a dreaded disease characterized by rapid development of hepatocellular dysfunction, leading to the failure of hepatic regeneration (1). Published studies have shown that the pathogenesis of FH is associated with huge liver infiltration of immune cells, which secrete a large number of pro-inflammatory cytokines and thus induce acute inflammatory necrosis of hepatocytes (2, 3). The bacterial infection has been considered as a key factor that contribute to the development of FH pathology (4). Indeed, mice injected with heat-killed *Propionibacterium acnes* followed by lipopolysaccharide (LPS), one of the most commonly used FH animal models, phenocopy the inflammatory infiltration in hepatic parenchyma and finally lead to the acute liver failure (5–8). Although the pathogenesis of FH has been extensively investigated, there is no proper therapeutic strategies for this disease, leading to high mortality if there is no supportive management and/or liver transplantation (9).

Myeloid derived suppressor cells (MDSC) are a heterogeneous group of immune cells derived from bone marrow and have been implicated to play important immunosuppressive and protective roles in human hepatitis, hepatocellular carcinoma or various mouse hepatitis models through different mechanism. For example, MDSC inhibited T cell proliferation and IFN-γ production in chronic HCV patients (10), and suppressed NK cell function during the pathogenesis of human hepatocellular carcinoma (11). In hepatitis mouse models, MDSC also exhibited immunosuppressive function through inhibiting the T cells proliferation, activation and secretion of pro-inflammatory cytokines, and thus protected against hepatic inflammation and fibrosis through different mechanisms (12–14). Therefore, increasing the number of MDSC in the liver may help to inhibit the occurrence of local inflammation of the liver and protect against FH. Indeed, administration of IL-25 dramatically prevented and reverses acute liver damage through promoting the recruitment of the MDSC into liver in FH mouse (15).

IL-25, also known as IL-17E, belongs to IL-17 cytokine family, and was initially found to be highly expressed in T helper (Th) 2 cells and promote the proliferation of Th2 cells and eosinophils (16–18). In addition, it has been reported that IL-25 exhibited inhibitory effect of the proliferation of Th1 and Th17 cells and further suppressed the occurrence of autoimmune diseases in mice (19, 20). However, it is not clear how IL-25 initiates the signal pathway to mediate MDSC recruitment into liver during FH pathogenesis. Published study has identified that IL-25 can bind to the heterodimer receptor composed of IL-17RA and IL-17RB, which then recruit Act1 to activate downstream NF-κB and MAPK (21–23), suggesting a similarity with IL-17A-induced signaling pathway. Our previous study has demonstrated that the serine/threonine protein kinase Tpl2 is a key component in regulating the IL-17A signaling pathway, in which the activated Tpl2 directly bound to and phosphorylated TAK1 and further induce the activation of downstream NF-κB and MAPK (24, 25). Based on the similarity of IL-17A- and IL-25-induced signaling and the critical protective role of IL-25 in FH, we speculated that Tpl2 may also regulated the FH pathogenesis through modulation of IL-25 signaling.

In the present study, we found that Tpl2 protected against FH-induced acute liver injury and mouse mortality. Loss of Tpl2 in hepatocytes suppressed IL-25-induced chemokine CXCL1/2 expression, which impaired the recruitment of MDSC into the liver, leading to promoted proliferation of liver-infiltrating CD4^+^ T cells and enhanced FH pathology.

## Results

### Tpl2 Protected Against *P. acne*/LPS-Induced FH

**Figure 1:**
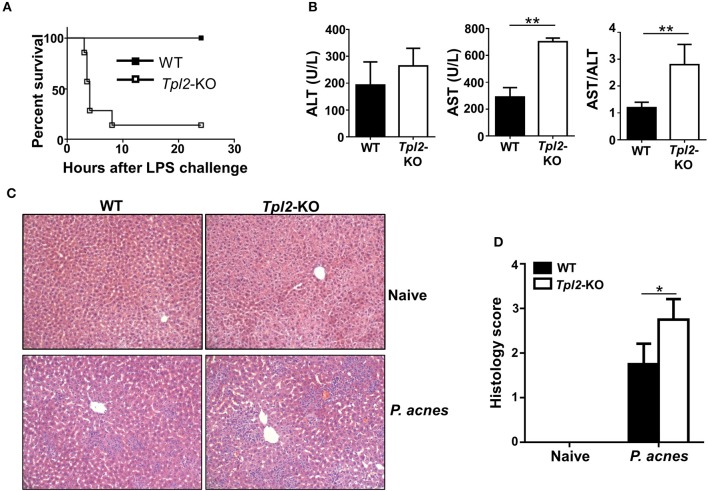
*Tpl2* deficiency exaggerated *P. acnes*/LPS-induced FH. WT and *Tpl2*-KO mice were injected with 0.5 mg *P. acnes* suspended in 200 μl of phosphate-buffered saline (PBS), and then 1 μg of LPS in 200 μl of PBS was injected on day 7 to induce fulminant hepatitis (FH). **(A)** Cumulative survival rates of WT and *Tpl2*-KO mice were analyzed (*n* = 7 mice/group) after LPS injection. **(B)** Serum levels of aminotransferase (ALT), aspartate aminotransferase (AST) and the AST/ALT ratios (*n* = 5 mice per group) were measured on day 7 after *P. acnes* priming. **(C)** H&E staining showing the representative inflammatory infiltration in the livers of WT and *Tpl2*-KO mice that injected with *P. acnes* at day 7. The liver sections from WT and *Tpl2*-KO naive mice were stained as negative controls (magnification, ×200). **(D)** Semiquantitative analysis of inflammatory conditions in the livers from WT and *Tpl2*-KO naive and *P. acnes*-primed mice. Results are mean ± SD from three independent experiments. Two-tailed Student's *t*-tests were performed. *P < 0.05; **P < 0.01.

To investigate the *in vivo* role of Tpl2 during FH pathogenesis, we induced a FH model by intravenously injecting the mice with heat-killed *P. acnes* and followed by LPS. In this model, only *P. acnes* priming is not lethal for the mice, and *P. acnes* priming plus LPS injection 7 days later will strongly induce acute liver damage, leading to FH-related mortality. However, *P. acnes* priming-induced liver inflammation is necessary and the reason for the mortality after LPS injection in this FH model (6, 7). As shown in Figure 1A, low dose of *P. acnes*/LPS priming provoked a non-lethal moderate hepatitis in wild-type (hereafter termed WT) mice. In contrast, *Tpl2*-deficient mice that induced with FH by using the same dose of *P. acnes*/LPS developed a much severer disease, leading to 86% lethality within 8 h (Figure 1A). Consistently, we observed the increased production of serum aspartate aminotransferase (AST) and higher ratio of AST/aminotransferase (ALT) levels, which is a hallmark of hepatitis-induced liver failure, in *Tpl2*-deficient mice accordingly (Figure 1B). In addition, histological analysis showed that there was more inflammatory infiltration observed in the *Tpl2*-deficient liver tissues on day 7 after *P. acnes* priming (Figures 1C,D). These results collectively suggested an important beneficial role of Tpl2 in protecting *P. acnes*/LPS-driven acute liver damage.

### *Tpl2* Deficiency Increased the Liver Infiltration of Pathogenic CD4^+^ T Cells

**Figure 2:**
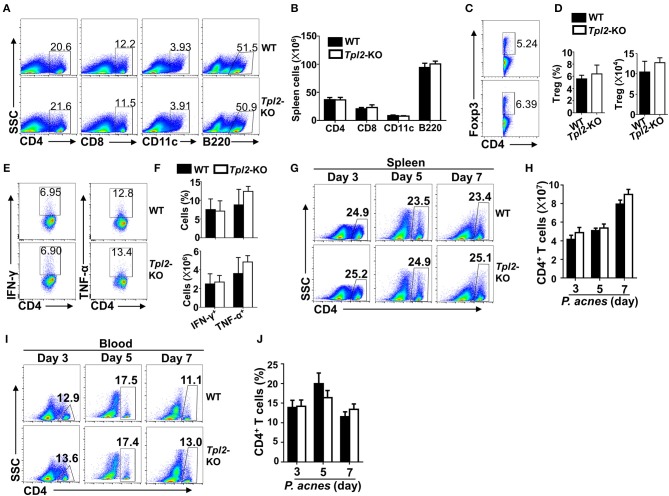
Tpl2 didn't affect peripheral immune activation during FH pathogenesis. The splenic cells or peripheral blood immune cells were isolated from *P. acnes*-primed WT and *Tpl2*-KO mice at day 3, 5, and 7 as described in Materials and methods (*n* = 4 mice/group), and subjected for flow cytometry analysis. **(A–F)** Flow cytometry analysis of the frequencies and absolute numbers of CD4^+^ T cells, CD8^+^ T cells, B220^+^ B cells, CD11c^+^ dendritic cells **(A,B)**, CD4^+^Foxp3^+^ Treg cells **(C,D)**, and IFN-γ- and TNF-α-producing pathogenic Th1 cells **(E,F)** in the spleens of WT and *Tpl2*-KO mice at day 7 after *P. acnes* priming. Data are presented as representative plots of the frequencies of immune cell subpopulations **(A,C,E)** and a summary graph of the cell frequencies or absolute cell numbers **(B,D,F)**. **(G–J)** Flow cytometry analysis of the frequencies and absolute numbers of CD4^+^ T cells in the spleens **(G,H)** or peripheral blood **(I,J)** of WT and *Tpl2*-KO mice at day 3, 5, and 7 after *P. acnes* priming. Data are presented as representative plots of the frequencies of immune cell subpopulations **(G,I)** and a summary graph of the cell frequencies or absolute cell numbers **(H,J)**. Results are mean ± SD from three independent experiments.

The exaggerated FH in *Tpl2*-deficient mice promoted us to examine the cellular mechanism by which Tpl2 protect against liver failure during FH pathogenesis. We firstly examined the peripheral immune activation after *P. acnes* priming, and the results revealed that *Tpl2*-deficient and WT control mice had similar frequencies and absolute numbers of CD4^+^ T cells, CD8^+^ T cells, CD11c^+^ dendritic cells, B220^+^ B cells and CD4^+^Foxp3^+^ regulatory T cells (Treg) in the spleens 7 days after challenged with *P. acnes* (Figures 2A–D). In addition, the frequencies and absolute numbers of IFN-γ- and TNF-α-producing T helper (Th)1 CD4^+^ T cells in the spleens were also comparable between the WT and *Tpl2*-deficient mice (Figures 2E,F). These data suggested Tpl2 does not affect peripheral immune activation during FH pathogenesis.

**Figure 3:**
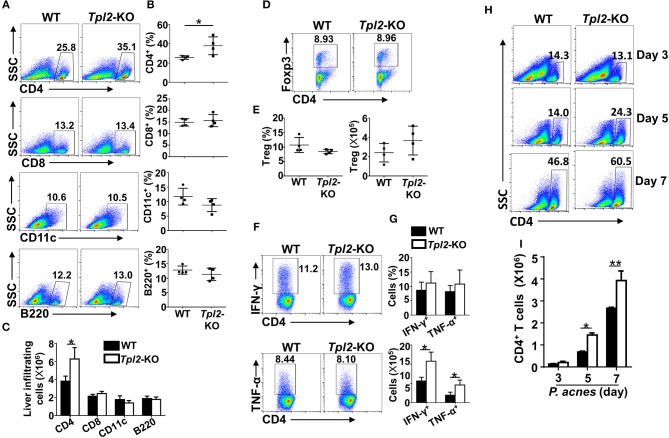
Tpl2 reduced liver infiltration of pathogenic CD4^+^ T cells. Liver-infiltrating immune cells were isolated from *P. acnes*-primed WT and *Tpl2*-KO mice at day 3, 5, and 7 as described in Materials and methods (*n* = 4 mice/group). **(A–G)** Flow cytometry analysis of the frequencies and absolute numbers of CD4^+^ and CD8^+^ T cells, B220^+^ B cell, CD11c^+^ dendritic cells **(A–C)**, CD4^+^Foxp3^+^ Treg cells **(D,E)**, and IFN-γ- and TNF-α-producing pathogenic Th1 cells **(F,G)** in the livers of WT and *Tpl2*-KO mice at day 7 after *P. acnes* priming. Data are presented as representative plots of the frequencies of immune cell subpopulations **(A,D,F)** and a summary graph of the frequencies and absolute cell numbers **(B,C,E,G)**. **(H,I)** Flow cytometry analysis of the frequencies and absolute numbers of CD4^+^ T cells in the livers of WT and *Tpl2*-KO mice at day 3, 5, and 7 after *P. acnes* priming. Data are presented as representative plots of the frequencies of immune cell subpopulations **(H)** and a summary graph of the absolute cell numbers **(I)**. Results are mean ± SD from three independent experiments. Two-tailed Student's *t*-tests were performed. *P < 0.05; **P < 0.01.

It is known that *P. acnes* priming promoted the liver infiltration of CD4^+^ T cells, which contributed to the inflammation-induced liver injury (8). Although the frequencies and absolute cell numbers of CD4^+^ T cells in the spleens or peripheral blood were comparable in WT and *Tpl2*-deficient mice during *P. acnes*-primed process (Figures 2G–J), loss of Tpl2 dramatically increased the frequencies and absolute numbers of CD4^+^ T cells in the livers as compared with that in WT liver, whereas didn't affect the liver infiltration of CD8^+^ T cells, dendritic cells, B cells and Treg cells at day 7 after *P. acnes* priming (Figures 3A–E). Since the Th1 cells are the major pathogenic contributor of *P. acnes*-induced liver injury (26), we next examined the TNF-α and IFN-γ production among the infiltrated CD4^+^ T cells. Interestingly, *Tpl2* deficiency didn't affect the ability of liver-infiltrating CD4^+^ T cells to produce TNF-α and IFN-γ, as reflected by comparable frequencies of IFN-γ- and TNF-α-producing Th1 cells in the inflamed livers of WT and *Tpl2*-deficient mice (Figures 3F,G). However, the absolute cell numbers of the pathogenic Th1 cells were dramatically increased in the inflamed livers of *Tpl2*-deficient mice (Figures 3F,G). Moreover, *Tpl2* deficiency gradually increased the liver infiltration of CD4^+^ T cells, notably at day 5 and 7 after *P. acnes* priming (Figures 3H,I). These data collectively suggested Tpl2 may inhibited the liver infiltration of CD4^+^ T cells during *P. acnes*-induced liver injury.

### Tpl2 Specifically Restricted the Proliferation of Liver-Infiltrating CD4^+^ T Cells

**Figure 4:**
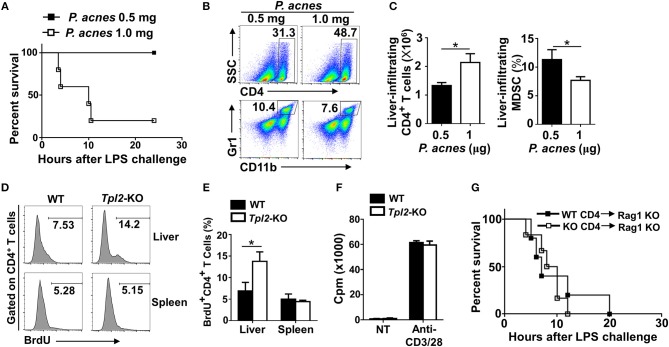
*Tpl2* deficiency specifically inhibited the proliferation of liver-infiltrated CD4^+^ T cells. **(A)** Cumulative survival analysis of WT C57BL/6 mice (*n* = 5 mice/group) that were injected with 0.5 or 1.0 mg *P. acnes* suspended in 200 μl of phosphate-buffered saline (PBS), and then with 1 μg of LPS in 200 μl of PBS at day 7 to induce fulminant hepatitis (FH). **(B,C)** Flow cytometry analysis of the frequencies and absolute numbers of CD4^+^ and Gr-1^+^CD11b^+^ MDSC in the livers of WT C57BL/6 mice at day 7 after different dose of *P. acnes* priming. Data are presented as representative plots of the frequencies of immune cell subpopulations **(B)** and a summary graph of the frequencies or absolute cell numbers **(C)**. **(D,E)** Flow cytometry analysis of CD4^+^ T cell proliferation in the spleens and livers of WT and *Tpl2*-KO mice at day 7 after *P. acnes* priming (*n* = 4 mice/group). Data are presented as a representative histogram **(D)** and a summary bar graph **(E)** showing the frequencies of proliferating BrdU^+^ CD4^+^ T cells. **(F)** Proliferation of WT and *Tpl2-*KO CD4^+^ T cells in the absence (NT) or presence of anti-CD3/CD28 antibodies, then assessed by [^3^H] thymidine incorporation. **(G)** Cumulative survival rates of age- and sex-matched *Rag1*-KO mice that adoptively transferred with WT and *Tpl2*-KO CD4^+^ T cells and then subjected to *P. acnes*/LPS-mediated FH induction (*n* = 5 mice/group). Results are mean ± SD from three independent experiments. Two-tailed Student's *t*-tests were performed. *P < 0.05.

To confirm the pathogenic role of liver-infiltrating CD4^+^ T cells after *P. acnes* priming in the FH model, we injected different dose of *P. acnes* and examined the survival rate and liver infiltration of CD4^+^ T cells. The results revealed that after challenged with a single shot of same dose of LPS, higher dose of *P. acnes* priming dramatically increased the mouse mortality rate as compared with that primed with lower dose of *P. acnes* (Figure 4A). As expected, higher dose of *P. acnes* priming significantly increased the infiltration of CD4^+^ T cells in the livers, along with decreased frequencies of liver infiltration of MDSC (Figures 4B,C), which is known to restrain the local inflammation in inflamed liver microenvironment through inhibiting T cell proliferation (15, 27). Therefore, we speculated that Tpl2 may regulate the proliferation of liver-infiltrating CD4^+^ T cells, and then injected bromodeoxyuridine (BrdU), a synthetic nucleoside that could be incorporated into newly synthesized DNA to monitor the cell proliferation, into WT or *Tpl2*-deficient mice before *P. acnes* priming. The flow cytometric analysis revealed that *Tpl2* deficiency specifically increased the frequencies of BrdU^+^ CD4^+^ T cells that isolated from the inflamed livers, whereas the frequencies of BrdU^+^ CD4^+^ T cells were comparable in the spleens between WT and *Tpl2*-deficient mice after *P. acnes* priming (Figures 4D,E), suggesting Tpl2 may indirectly regulate the proliferation of CD4^+^ T cells in the inflamed liver microenvironment. Indeed, Tpl2 is dispensable for the *in vitro* CD4^+^ T cell proliferation, as characterized by comparable proliferation rate of naïve WT and *Tpl2*-deficient CD4^+^ T cells upon the stimulation of anti-CD3 plus anti-CD28 antibodies (Figure 4F).

To further exclude the possibility that Tpl2 may directly function in CD4^+^ T cell to regulate FH pathogenesis, we adoptively transferred WT or *Tpl2*-deficient CD4^+^ T cells into T cell-deficient *Rag1*-KO mice, which were then induced the FH model by injecting *P. acnes*/LPS. Expectedly, the FH-induced mortality rates are comparable in recipient *Rag1*-KO mice that either transferred with WT or *Tpl2*-deficient CD4^+^ T cells (Figure 4G). Collectively, these results suggested that Tpl2 specifically restricted the proliferation of liver-infiltrating CD4^+^ T cells through an indirect mechanism during FH pathogenesis.

### Tpl2 Mediated the Recruitment of MDSC Into Liver

**Figure 5:**
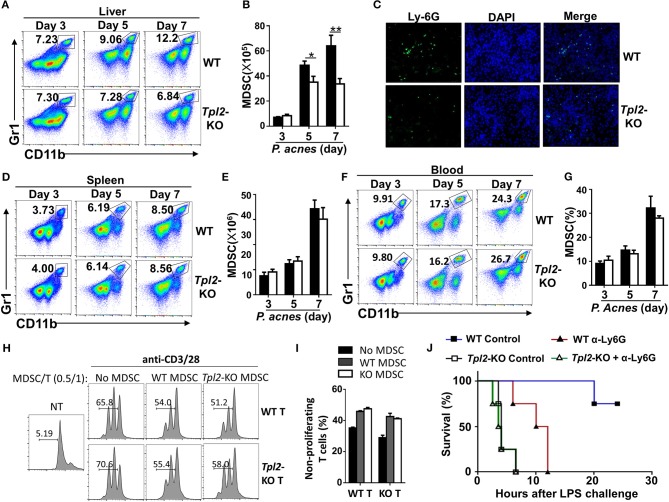
Tpl2 mediated the liver recruitment of MDSC during FH pathogenesis. **(A,B,D–G)** Flow cytometric analysis of Gr-1^+^CD11b^+^ MDSC in the livers **(A,B)** or spleens **(D,E)** or peripheral blood **(F,G**) of WT and *Tpl2*-KO mice at day 3, 5, and 7 after *P. acnes* priming (*n* = 4 mice/group). Data are presented as representative plots **(A,D,F)** and summary bar graph **(B,E,G)** showing the absolute numbers or frequencies of MDSC. **(C)** Immunofluorescence images showing the infiltrated MDSC by using the anti-Ly6G antibody in the liver sections obtained from WT and *Tpl2*-KO mice at day 7 after *P. acnes* priming (magnification, ×200). **(H,I)** Flow cytometry analysis of the proliferation of WT CD4^+^ T cells that labeled with CFSE, and then cocultured with WT or *Tpl2*-KO MDSC at the indicated ratio in the absence (NT) or presence of anti-CD3/28 antibodies for 72 h. Data are presented representative histograms **(H)** and bar graph **(I)**. **(J)** Cumulative survival rates of WT and *Tpl2*-KO mice that injected with anti-Ly6G antibody (200 μg/mouse, three times) to deplete *in vivo* MDSC or control antibody, and then subjected to *P. acnes*/LPS-mediated FH induction (*n* = 4 mice/group). Results are mean ± SD from three independent experiments. Two-tailed Student's *t*-tests were performed. *P < 0.05; **P < 0.01.

Considering the indirect function of Tpl2 in regulating CD4^+^ T cell proliferation, we examined the infiltration status of MDSC in inflamed liver. In contrast to the increased infiltrating rate of CD4^+^ T cells in inflamed liver (Figures 3H,I), *Tpl2* deficiency gradually decreased the frequencies and absolute numbers of liver-infiltrating CD11b^+^Gr-1^+^ MDSC, notably at day 5 and 7 after *P. acnes* priming, as compared with that of WT mice (Figures 5A,B). The immunofluorescence analysis also confirmed the decreased infiltration of MDSC in the hepatic parenchyma of *Tpl2*-KO mice at day 7 after *P. acnes* priming (Figure 5C). However, loss of Tpl2 neither altered the frequencies and absolute numbers of MDSC in the spleens nor affected the peripheral distribution of MDSC in the circulation system during *P. acnes*-primed process (Figures 5D–G). In addition, *in vitro* proliferation assay revealed that *Tpl2*-deficient MDSC exhibited similar ability as WT MDSC to suppress either WT or *Tpl2*-deficient T cell proliferation after cocultured with CD4^+^ T cell that stimulated with anti-CD3 plus anti-CD28 antibodies (Figures 5H,I). These data suggested that *Tpl2* deficiency suppressed the liver recruitment of MDSC without affecting their immunosuppressive function.

We next examined whether the impaired liver recruitment of MDSC contributed to the enhanced mortality in *Tpl2*-deficient FH mice. To this end, we specifically deleted the MDSC by injection of anti-Ly-6G neutralizing antibody (Supplementary Figures 1A,B), and challenged mice with *P. acnes*/LPS to induce FH. As expected, *in vivo* MDSC depletion dramatically increased the mortality rate of WT FH mice, and largely abolished the difference of the survival rate between WT and *Tpl2*-deficient mice that injected with *P. acnes*/LPS (Figure 5J). These results collectively established Tpl2 as a critical mediator of MDSC mobilization into liver to protect inflammation-induced liver injury during FH pathogenesis.

### Tpl2 Functioned in Liver-Resident Cells to Protect Against FH

**Figure 6:**
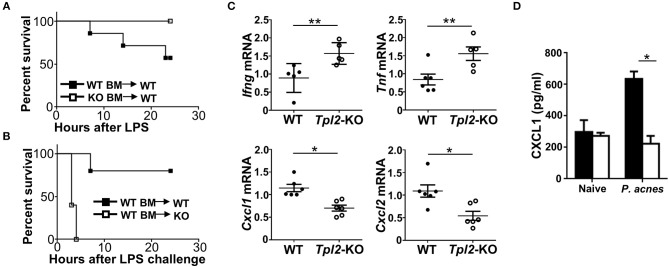
Tpl2 functioned in liver-resident cells to protect against FH. **(A,B)** Cumulative survival rates of the lethally irradiated bone marrow (BM) chimeric WT mice that reconstituted with WT or *Tpl2*-KO BM **(A)** or the BM chimeric WT or *Tpl2*-KO mice that reconstituted with WT BM **(B)**, and then subjected to *P. acnes*/LPS-mediated FH induction (*n* = 7 or 5 mice/group). **(C)** QPCR analysis to determine the relative mRNA expression level of proinflammatory genes in livers of WT and *Tpl2*-KO mice at day 3 after *P. acnes* priming (*n* = 6 mice/group). Data were normalized to a reference gene, *Actb*. **(D)** Enzyme-linked immunosorbent assay of CXCL1 cytokine secretion in the supernatants of liver homogenates from WT and *Tpl2*-KO naïve or *P. acnes*-primed mice at day 3 (*n* = 4). Results are mean ± SD from three independent experiments. Two-tailed Student's *t*-tests were performed. *P < 0.05; **P < 0.01.

To figure out which type of cells *in vivo* was directly targeted by Tpl2 to protect against FH-induced liver failure, we generated the mixed bone marrow (BM) chimeric mice by reconstituting the lethally irradiated WT mice with WT or *Tpl2*-deficient BM, which were then challenged with *P. acnes*/LPS to induce FH. Unexpectedly, *Tpl2*-deficient BM reconstituted chimeric mice were totally resistant to FH-induced death (Figure 6A), suggesting that *Tpl2* deficiency in myeloid cells (including macrophages and MDSC) does not contribute to the aggregation of FH-induced mortality. However, when reconstituting the WT BM into lethally irradiated WT or *Tpl2*-deficient mice, we found that WT BM failed to induce FH-mediated death in WT recipient mice, whereas dramatically promoted the mortality rate of *Tpl2*-deficient recipient mice (Figure 6B). These data collectively suggested that Tpl2 didn't target myeloid cells, but functioned in liver-resident cells to protect against FH pathology.

Next, we examined the proinflammatory cytokine and chemokine induction in the livers at the early priming phase of FH model. After 3 days of *P. acnes* challenge, the expression of Th1 cytokine genes *Ifng* and *Tnf* in the *Tpl2*-deficient inflamed livers were much higher than that in WT livers (Figure 6C), which suggested that the increased liver infiltration of pathogenic Th1 cells as shown in Figure 3G may contribute to the enhanced expression of these pro-inflammatory genes. Accordingly, the expression of MDSC-attracting chemokine genes *Cxcl1* and *Cxcl2* were dramatically suppressed in the *Tpl2*-deficient livers as compared with that in WT livers (Figure 6C). In addition, the expression of the genes that encoding DC-recruiting chemokine MIP-1α (6) and other two MDSC-attracting chemokine CCL17 and CCL19 (15) were not affected in the livers of *P. acnes*-primed *Tpl2*-deficient mice (Supplementary Figures 1C,D). Moreover, the enzyme-linked immunosorbent assay confirmed that *Tpl2* ablation inhibited *P. acnes*-induced CXCL1 chemokine protein production in the liver parenchyma as compared with that in WT livers (Figure 6D). Together, these results suggested that Tpl2 directly functioned in liver-resident cells, but not in peripheral immune cells, to mediate MDSC recruitment, and thus protect against FH pathology.

### Tpl2 Regulated IL-25 Signaling in Hepatocytes

**Figure 7:**
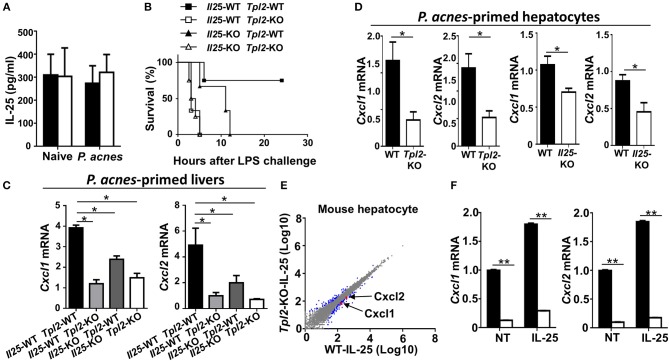
Tpl2 regulated IL-25 signaling in hepatocytes. **(A)** Enzyme-linked immunosorbent assay showing IL-25 cytokine secretion in the supernatants of liver homogenates from WT and *Tpl2*-KO naïve or *P. acnes*-primed mice at day 3 (*n* = 3 or 4). **(B)** Cumulative survival rates of WT, *Tpl2*-KO, *Il25*-KO, *Tpl2*/*Il25* double knockout mice that subjected to *P. acnes*/LPS-mediated FH induction (*n* = 3 or 4 mice/group). **(C,D)** QPCR analysis of *Cxcl1* and *Cxcl2* genes' expression of livers **(C)** or hepatocytes **(D)** in WT, *Tpl2*-KO, *Il25*-KO, *Tpl2*/*Il25* double knockout mice at day 3 after *P. acnes* priming (*n* = 6 mice/group). **(E)** Scatter diagram showing the RNA-sequencing analysis of the gene expression pattern in WT and *Tpl2*-KO primary hepatocytes that stimulated by IL-25 for 8 h. The blue dots indicated the IL-25-induced up- or down-regulated genes, and the red dots indicated the down-regulation of *Cxcl1* and *Cxcl2* in *Tpl2*-KO hepatocytes. **(F)** QPCR determining the relative mRNA expression level of *Cxcl1* and *Cxcl2* in WT and *Tpl2*-KO hepatocytes that left untreated (NT) or stimulated with IL-25 for 8 h. Data were normalized to a reference gene, *Actb*. Results are mean ± SD from three independent experiments. Two-tailed Student's *t*-tests were performed. *P < 0.05; **P < 0.01.

Published study has suggested that IL-25 is highly produced in both human and mouse livers, and it is critical for the liver recruitment of MDSC in D-Gal/LPS-induced FH mouse model (15). In addition, we have previously demonstrated that Tpl2 mediates the activation of signaling pathway induced by IL-17A, which belongs to the same IL-17 family as IL-25 (24, 25). Therefore, we speculated that Tpl2 may potentially modulates IL-25 signaling in the liver-resident cells to regulate FH pathogenesis. To test this hypothesis, we firstly examined the IL-25 production in the livers, and found that there is no significant difference of IL-25 levels in the liver homogenates between WT and *Tpl2*-deficient mice that were either under physiological condition or challenged with *P. acnes* (Figure 7A), suggesting Tpl2 is dispensable for the IL-25 secretion in the liver tissue. In addition, we observed that IL-25 production in the livers of *P. acnes*-primed mice were comparable with that of naïve mice (Figure 7A), which is different from the D-Gal/LPS-induced FH model that IL-25 levels are decreased in the livers of FH mice (15). Next, we generated the *Tpl2*/*Il25* double knockout mice and examined the potential *in vivo* link between Tpl2 and IL-25. Expectedly, IL-25 deletion under WT or *Tpl2*-KO background both dramatically increased the mortality rate of FH mice, and suppressed the expression of MDSC-recruiting chemokine genes *Cxcl1* and *Cxcl2* in *P. acnes*-primed livers (Figures 7B,C), implying IL-25 is also critical for the liver recruitment of MDSC and thus protect against *P. acnes*/LPS-induced FH. Interestingly, *Tpl2*/*Il25* double knockout mice didn't further exaggerated the disease severity of *P. acnes*/LPS-induced FH when compared with *Tpl2*-deficient mice, as reflected by comparable mortality rate of these two strains of FH mice (Figure 7B), suggesting Tpl2-mediated prevention of FH is indeed through IL-25 signaling.

Next, we examined the cellular source of CXCL1 and CXCL2 in the *P. acnes*-primed livers. The results revealed that *Tpl2* deficiency didn't affect the expression of *Cxcl1* and *Cxcl2* in *P. acnes*-primed liver CD11b^+^F4/80^+^GR-1^−^ kuffer cells and macrophages (Supplementary Figure 1E). However, loss of Tpl2 or IL-25 both significantly suppressed these two genes' expression in the hepatocytes that isolated from *P. acnes*-primed livers (Figure 7D). In addition, the RNA sequencing analysis showed that the expression of *Cxcl1, Cxcl2*, and other IL-25-response genes are dramatically decreased in IL-25-stimulated *Tpl2*-deficient primary mouse hepatocytes as compared with that of WT cells (Supplementary Figures 2A–D, Figure 7E). Moreover, the quantitative PCR resulted also confirmed that Tpl2 is indispensable for the constitutively and IL-25-induced expression of *Cxcl1* and *Cxcl2* in primary mouse hepatocytes (Figure 7F). Collectively, these results suggested that Tpl2 mediated IL-25 signaling in hepatocyte to protect against FH.

## Discussion

FH is a life-threatening disease and liver transplantation is the only definitive treatment for the acute liver injury. However, the obvious side-effects of transplantation, such as donor shortage, immune rejection, detrimental effect of immunosuppressive drugs, etc., suggested an urgent to develop novel therapeutic strategies (1, 9, 28). Recently, accumulating evidences suggested that MDSC is critical to maintain the immunosuppressive niche in inflamed liver during the pathogenesis of various kinds of human hepatitis and related mouse models, and increased infiltration of MDSC effectively attenuated the liver inflammation and protected FH-induced acute liver failure (10–15, 27). However, the molecular mechanism through which driving MDSC mobilization into inflamed liver remain elusive. Here we identified the protein kinase Tpl2 as an essential mediator to mobilize MDSC into liver during FH pathogenesis, and thus Tpl2 effectively protected the mice against FH-induced acute liver failure and mortality. Therefore, Tpl2 may have therapeutic potential for the treatment of FH.

Tpl2 is a protein kinase that was initially identified as protooncogene due to the tumor promoting function of its C-terminal truncation (29, 30). The expression of Tpl2 is universal and it is found to expressed in both innate and adaptive immune cells and in diverse tissues, including the liver, lung, and intestines (30–33). The immune-regulatory function of Tpl2 is largely attributed to its activation of the MEK/ERK pathway in toll-like receptor (TLR), interleukin-1 receptor (IL-1R), or tumor necrosis factor receptor (TNFR) signaling (34, 35). In addition, Tpl2 also modulate the activation of p38, JNK, protein kinase B, and mammalian target of rapamycin in a context-dependent manner (25, 36). We previously found that Tpl2 functions in astrocytes to mediate IL-17A-induced chemokine (*Cxcl1/2*) expression through promoting TAK1 phosphorylation and its downstream NF-κB, p38, and JNK activation, whereas ERK activation is not affected (24). Therefore, it is not surprising we found in the present study that Tpl2 functioned in hepatocyte to modulate *Cxcl1/2* expression, which then modulated the recruitment of MDSC into liver during FH pathogenesis. A recent study has suggested that Tpl2 exhibited neutrophil intrinsic function to mediate the trafficking of this type of immune cells (37), so it is also possible that Tpl2 may functioned directly in MDSC to promote its liver mobilization in FH mice. However, the increased mortality was only observed in Tpl2 germline knockout FH mice or *Tpl2*-deficient recipient chimeric FH mice that adoptively transferred with WT BM, but not in the WT recipient chimeric FH mice that adoptively transferred with either WT or *Tpl2*-deficient BM, suggesting the liver MDSC mobilization during FH pathogenesis is not attributed to the direct intrinsic function of Tpl2 in MDSC, but in hepatocytes.

Although IL-25 is one of the IL-17 family protein, there is no functional similarity of IL-25 as compared with the pro-inflammatory IL-17A (16, 17). For example, IL-25 augments type 2 immune responses and promote the airway inflammation of patients with asthma (38). A recent study suggested that IL-25 is highly expressed in both human and mouse liver, and plays a critical function in maintaining the homeostasis and limiting local inflammation through recruiting the immunosuppressive MDSC (15). Nevertheless, the molecular mechanism through which IL-25 recruit MDSC into liver is not clear. Our present study provided a Tpl2 link between IL-25 and MDSC mobilization, and established Tpl2 as a key mediator of IL-25-induced signaling that contribute to the MDSC recruitment. In addition, during *P. acnes*/LPS-induced FH pathogenesis, Tpl2 seemed specifically mediate IL-25-induced expression of CXCL1/2, but not affected the induction of CCL17, a previously reported MDSC-attracting chemokine that could be induced by IL-25 administration in D-Gal/LPS-induced FH mice (15), suggesting Tpl2 modulated IL-25-induced chemokine expression in a context-dependent manner.

Our previously study has suggested that Tpl2 critically regulate IL-17A-induced signaling in astrocytes to mediate autoimmune inflammation, here we also demonstrated that Tpl2 is a key modulator in IL-25-induced signaling in hepatocyte to restrain hepatitis. This functional controversy may be due to Tpl2 regulates the function of different cells upon different stimulus, and suggest Tpl2 have a dual role in promoting or restraining inflammatory processes in a context-dependent manner.

**Figure 8:**
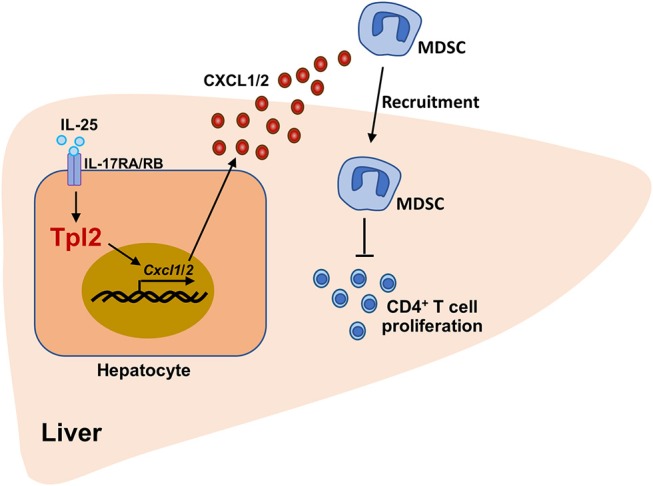
The working model of Tpl2 in protecting against fulminant hepatitis. During the pathogenesis of *P. acnes*/LPS-induced FH, high levels of IL-25 in the liver microenvironment activated the signaling pathway mediated by IL-17RA/IL-17RB heterodimer receptor in hepatocytes, which were induced the expression of *Cxcl1/2* on a Tpl2-dependent manner. Increased CXCL1/2 production promoted the liver recruitment of the immunosuppressive MDSC, which further impaired the proliferation of liver-infiltrated pathogenic CD4^+^ T cells, and finally suppressed the inflammation-induced acute liver injury.

In conclusion, our findings demonstrated that Tpl2 effectively attenuated the severity of acute liver injury and increased the survival rate of FH mice. Mechanistically, Tpl2 functioned in hepatocytes to mediate IL-25-induced CXCL1/2 chemokines, which promoted the recruitment of MDSC to suppress Th1-mediated local inflammation, resulting in the amelioration of FH (Figure 8). Our data not only highlighted a novel function of Tpl2 in mediating IL-25 signaling, but also raised the possibility to develop Tpl2-based therapeutic strategies against this dreaded disease.

## Materials and Methods

### Mice

*Tpl2*-deficient mice (C57BL/6 background) were described as previously (24). The *Tpl2*^+/−^ mice were bred to generate age-matched *Tpl2*^−/−^ (*Tpl2*-KO) and *Tpl2*^+/+^ (WT) mice. The *Il25*-defcient mice (C57BL/6 background) were provided by Dr. Y. Qian (Shanghai Institutes for Biological Sciences, Chinese Academy of Sciences). In some experiments, *Tpl2*^−/−^ mice were crossed with *Il25*^−/−^ mice to generate *Tpl2*^+/−^*Il25*^+/−^ mouse, which were then bred to generate age-matched *Tpl2*^+/+^*Il25*^+/+^, *Tpl2*^−/−^*Il25*^+/+^, *Tpl2*^+/+^*Il25*^−/−^, and *Tpl2*^−/−^*Il25*^−/−^ mice. *Rag1*^−/−^ mice (NM-KO-00069) were purchased from Shanghai Model Organisms Center. Mice were maintained in a specific pathogen-free facility, and all animal experiments were in accordance with protocols approved by the institutional Biomedical Research Ethics Committee, Shanghai Institutes for Biological Sciences, Chinese Academy of Sciences.

### Induction of FH Mouse Model

For the induction of FH model, the age- and sex-matched mice were intravenously injected with 0.5 mg of heat-killed *P. acnes* suspended in 200 μl of phosphate-buffered saline (PBS) and after 7 days mice were injected intravenously with 1 μg of LPS and were monitored the survival rate. In some experiments, the WT and *Tpl2*-deficient mice were intravenously injected with 200 μg anti-Ly6G antibody for 3 times to deplete MDSC *in vivo*, or the *Rag1*-KO mice were adoptively transferred with WT or *Tpl2*-deficient CD4^+^ T cells, or the lethally irradiated mice that were reconstituted with WT or *Tpl2*-deficient bone marrows, and then these mice were injected with *P. acnes*/LPS to induce FH and were monitored the survival rate.

### Antibodies and Reagents

APC conjugated anti-mouse CD4 (17-0041-83), PB conjugated anti-mouse CD4 (48-0042-82), PE-cy7 conjugated anti-mouse CD8 (25-0081-82), APC-cy7 conjugated anti-mouse CD11b (47-0112-82), PB conjugated anti-mouse CD11c (48-0114-82), PerCP conjugated anti-mouse Ly6G (46-9668-82), FITC conjugated anti-mouse B220 (11-0452-85), PE conjugated anti-mouse CD45 (12-0451-83), APC conjugated anti-mouse IFNγ (17-7311-82), PE conjugated anti-mouse TNFα (12-7321-82), PE-cy7 conjugated anti-mouse CD25 (25-0251-82), APC conjugated anti-mouse Foxp3 (17-5773-82), PE conjugated anti-mouse CD44 (12-0441-83), FITC conjugated anti-mouse CD62L (11-0621-86), anti-mouse CD3 (16-0031-86), and anti-mouse CD28 (16-0281-86) antibodies were purchased from eBioscience. BrdU Flow Kits (559619) were purchased from BD Biosciences. Anti-mouse Ly6G (BE0075) antibody was purchased from Bioxcell. Alexa Fluor 488-conjugated anti-Rat IgG secondary antibody (A-21210) was from Thermo Fisher. Mouse anti-CD4 (L3T4, 130-049-201) and mouse anti-Ly-6G (130-092-332) Micro Beads were purchased from Miltenyi Biotec. Murine IL-25 (1399) were purchased from R&D. Lipopolysaccharides (LPS, L3129) were purchased from Sigma. *P. acnes* were prepared as previously described (7).

### Flow Cytometry

The infiltrated immune cells from WT and *Tpl2*-deficient inflamed livers were prepared through 33% Percoll gradient as previously described (6). The collected liver-infiltrated immune cells or splenic cell suspensions were stained with the indicated antibodies and were subjected to flow cytometry analyses as previously described by using a Beckman Gallios flow cytometer (39). For the intracellular staining of TNF-α, IFN-γ, and Foxp3, the cells were fixed and permeabilized by fixation/permeabilization buffer (Thermo Fisher) before staining these antibodies, and then detected by flow cytometer. The absolute numbers of splenic and liver-infiltrating immune cells subpopulations were calculated based on their frequencies and the total number of isolated splenic and liver immune cells, and the data were presented as the average numbers of immune cell subpopulations per one spleen or liver of one mouse.

### Histology and Immunofluorescence Analysis

Liver specimens were fixed in 4% paraformaldehyde and paraffin-embedded. Deparaffinized sections (8 μm) were stained with hematoxylin and eosin. Semiquantitative analysis of the status of liver inflammation was performed in a blinded manner as previously described (40). Briefly, the H&E stained liver slides were scored by a pathologist in a “blinded fashion” to determine the degree of inflammatory condition as follows: 0 = no infiltration, 1 = minimal/slight infiltration, 2 = moderate infiltration, 3 = severe infiltration. For immunofluorescence staining, the frozen sections (10 μm) from liver specimens were incubated with rat anti-mouse Ly6G (BE0075, Bioxcell) and were then labeled with Alexa Fluor 488-conjugated rabbit anti-rat IgG (A21210, Invitrogen), and the nuclei were stained by using DAPI (28718-90-3, Sangon Biotech).

### Bone Marrow Chimeras

The bone marrow cells were prepared from WT or *Tpl2*-deficient mice and adoptively transferred into lethally irradiated (^137^Cs, γ-ray, 950 rad) WT or *Tpl2*-deficient mice (around 7-week-old; 10^7^ cells/mouse) as previously described (41). The lethal-dose irradiation would eliminate the bone marrow and peripheral immune cells without affecting the radioresistant liver-resident cells, and the bone marrow chimeric mice would thus have their peripheral immune system reconstituted. After 8 weeks, the chimeric mice were applied for the indicated experiments.

### Mouse Hepatocyte Isolation

The mouse primary hepatocytes were prepared as previously described (42). In brief, the livers were sequentially perfused with Earle's balanced salt solutions (EBSS) without Ca^+^ and Mg^+^ containing EGTA, EBSS with Ca^+^ and Mg^+^ containing Hepes, EBSS with Ca^+^ and Mg^+^ containing Hepes and Collagenase IV, and the liver cells were squeezed out to obtain cell suspension in DMEM medium, which were then applied for centrifugation over a mixture of 9 ml Percoll, 1 ml 10 × EBSS and 10 ml DMEM. The precipitated hepatocytes were suspended and cultured with DMEM complete medium in a collagen-coated culture dish. Cell viability was determined by using Trypan blue exclusion assay.

### *In vivo* BrdU Incorporation

Seven days after *P. acnes* priming, 2 mg BrdU (559619, BD) in 200 μl PBS was intraperitoneally injected into WT or *Tpl2*-deficient mice. The mice were sacrificed 2 h after the BrdU administration, and the immune cell suspensions from livers or spleens were prepared for flow cytometric analysis.

### T Cell Proliferation Assay

The WT CD4^+^ T cells were purified by MACS sorting and were labeled with 5 μM CFSE. The labeled cells were then seeded in the anti-CD3/CD28 antibodies-pre-coated plates and cocultured with MDSCs that isolated from WT or *Tpl2*-deficient bone marrow at the indicated ratio for 72 h. The cell proliferation was then determined by flow cytometry. In some experiment, WT or *Tpl2*-deficient CD4^+^ T cells were seeded in anti-CD3/CD28 antibodies-pre-coated plates with 3 replicates and cultured for a total 72 h. The cell proliferation was recorded based on the [^3^H] thymidine labeling 8 h before examination.

### RNA-Seq Analysis

Total RNA isolated from WT and *Tpl2*-KO hepatocytes stimulated with IL-25 were subjected to RNA-sequencing analysis. RNA sequencing was performed by BGI Tech Solutions. Transcriptomic reads from the RNA-Seq experiments were mapped to a reference genome (build mm 10) by using Bowtie. Gene expression levels were quantified by using the RSEM software package. Significant genes were defined by the *p*-value and false discovery rate of cutoff of 0.05 and fold changes ≥1.5. Differentially expressed genes were analyzed by the IPA and DAVID bioinformatics platform.

### Quantitative RT-PCR

Liver tissues or cell samples were homogenized in Trizol reagent (Invitrogen). The cDNA was synthesized from 500 ng of extracted total RNA using M-MLV Reverse Transcriptase kit (Takara) according to the manufacturer's instructions. Quantitative PCR was performed with SYBR-Green premix ExTaq (Roche) and detected by a Real-time PCR System by using gene-specific primers. Gene expression was assessed in triplicate and normalized to a reference gene, β-actin. The gene-specific PCR primers are listed in Supplementary Table 1.

### Quantification and Statistical Analysis

Statistical analyses were measured by GraphPad Software. Except where otherwise indicated, all the presented data are representative results of at least three independent repeats. Data are presented as mean ± SD, and the *P*-values were determined by two-tailed Student's *t*-tests. The *P*-values <0.05 were considered statistically significant.

## Data Availability

The RNA-Sequencing data have been deposited into the Gene Expression Omnibus (accession code GSE125764). All other data supporting the findings of this study are available from the corresponding author on reasonable request.

## Ethics Statement

This study was carried out in accordance with the recommendations of animal protocols that approved by Biomedical Research Ethics Committee, Shanghai Institutes for Biological Sciences, Chinese Academy of Sciences. The protocol was approved by the Biomedical Research Ethics Committee, Shanghai Institutes for Biological Sciences, Chinese Academy of Sciences.

## Author Contributions

JX designed and performed the experiments, prepared the figures, and wrote part of the manuscript. SP, YW, and JL contributed to part of the experiments. YQ provided the *Il25*-deficient mice. MH and YZ supervised the work and contributed to data analysis. YX designed and supervised the work, prepared the figures, and wrote the manuscript.

### Conflict of Interest Statement

The authors declare that the research was conducted in the absence of any commercial or financial relationships that could be construed as a potential conflict of interest.

## References

[1] Bernal W Auzinger G Dhawan A Wendon J. Acute liver failure. Lancet. (2010) 376:190–201. 10.1016/S0140-6736(10)60274-7 20638564

[2] Antoniades CG Berry PA Wendon JA Vergani D. The importance of immune dysfunction in determining outcome in acute liver failure. J Hepatol. (2008) 49:845–61. 10.1016/j.jhep.2008.08.009 18801592

[3] Crispe IN. The liver as a lymphoid organ. Annu Rev Immunol. (2009) 27:147–63. 10.1146/annurev.immunol.021908.132629 19302037

[4] Rolando N Harvey F Brahm J Philpott-Howard J Alexander G Gimson A. Prospective study of bacterial infection in acute liver failure: an analysis of fifty patients. Hepatology. (1990) 11:49–53. 10.1002/hep.1840110110 2295471

[5] Nakayama Y Shimizu Y Hirano K Ebata K Minemura M Watanabe A. CTLA-4Ig suppresses liver injury by inhibiting acquired immune responses in a mouse model of fulminant hepatitis. Hepatology. (2005) 42:915–24. 10.1002/hep.20872 16175605

[6] Xiao Y Xu J Mao C Jin M Wu Q Zou J. 18Beta-glycyrrhetinic acid ameliorates acute Propionibacterium acnes-induced liver injury through inhibition of macrophage inflammatory protein-1alpha. J Biol Chem. (2010) 285:1128–37. 10.1074/jbc.M109.037705 19897483 PMC2801241

[7] Zhang Y Cai W Huang Q Gu Y Shi Y Huang J. Mesenchymal stem cells alleviate bacteria-induced liver injury in mice by inducing regulatory dendritic cells. Hepatology. (2014) 59:671–82. 10.1002/hep.26670 23929707 PMC4298763

[8] Zhang Y Yoneyama H Wang Y Ishikawa S Hashimoto S Gao JL. Mobilization of dendritic cell precursors into the circulation by administration of MIP-1alpha in mice. J Natl Cancer Inst. (2004) 96:201–9. 10.1093/jnci/djh024 14759987

[9] Nemes B Gelley F Piros L Zadori G Gorog D Fehervari I. The impact of Milan criteria on liver transplantation for hepatocellular carcinoma: first 15 years' experience of the Hungarian Liver Transplant Program. Transplant Proc. (2011) 43:1272–4. 10.1016/j.transproceed.2011.03.077 21620108

[10] Cai W Qin A Guo P Yan D Hu F Yang Q. Clinical significance and functional studies of myeloid-derived suppressor cells in chronic hepatitis C patients. J Clin Immunol. (2013) 33:798–808. 10.1007/s10875-012-9861-2 23354838

[11] Hoechst B Voigtlaender T Ormandy L Gamrekelashvili J Zhao F Wedemeyer H. Myeloid derived suppressor cells inhibit natural killer cells in patients with hepatocellular carcinoma via the NKp30 receptor. Hepatology. (2009) 50:799–807. 10.1002/hep.23054 19551844 PMC6357774

[12] Cripps JG Wang J Maria A Blumenthal I Gorham JD. Type 1 T helper cells induce the accumulation of myeloid-derived suppressor cells in the inflamed Tgfb1 knockout mouse liver. Hepatology. (2010) 52:1350–9. 10.1002/hep.23841 20803559 PMC2947571

[13] Jenne CN Wong CH Zemp FJ McDonald B Rahman MM Forsyth PA. Neutrophils recruited to sites of infection protect from virus challenge by releasing neutrophil extracellular traps. Cell Host Microbe. (2013) 13:169–80. 10.1016/j.chom.2013.01.005 23414757

[14] Suh YG Kim JK Byun JS Yi HS Lee YS Eun HS. CD11b(+) Gr1(+) bone marrow cells ameliorate liver fibrosis by producing interleukin-10 in mice. Hepatology. (2012) 56:1902–12. 10.1002/hep.25817 22544759 PMC3427419

[15] Sarra M Cupi ML Bernardini R Ronchetti G Monteleone I Ranalli M. IL-25 prevents and cures fulminant hepatitis in mice through a myeloid-derived suppressor cell-dependent mechanism. Hepatology. (2013) 58:1436–50. 10.1002/hep.26446 23564603

[16] Angkasekwinai P Park H Wang YH Wang YH Chang SH Corry DB. Interleukin 25 promotes the initiation of proallergic type 2 responses. J Exp Med. (2007) 204:1509–17. 10.1084/jem.20061675 17562814 PMC2118650

[17] Fort MM Cheung J Yen D Li J Zurawski SM Lo S. IL-25 induces IL-4, IL-5, and IL-13 and Th2-associated pathologies *in vivo*. Immunity. (2001) 15:985–95. 10.1016/S1074-7613(01)00243-6 11754819

[18] Kim MR Manoukian R Yeh R Silbiger SM Danilenko DM Scully S. Transgenic overexpression of human IL-17E results in eosinophilia, B-lymphocyte hyperplasia, and altered antibody production. Blood. (2002) 100:2330–40. 10.1182/blood-2002-01-0012 12239140

[19] Caruso R Sarra M Stolfi C Rizzo A Fina D Fantini MC. Interleukin-25 inhibits interleukin-12 production and Th1 cell-driven inflammation in the gut. Gastroenterology. (2009) 136:2270–9. 10.1053/j.gastro.2009.02.049 19505427

[20] Liu D Cao T Wang N Liu C Ma N Tu R. IL-25 attenuates rheumatoid arthritis through suppression of Th17 immune responses in an IL-13-dependent manner. Sci Rep. (2016) 6:36002. 10.1038/srep36002 27812008 PMC5095710

[21] Gu C Wu L Li X. IL-17 family: cytokines, receptors and signaling. Cytokine. (2013) 64:477–85. 10.1016/j.cyto.2013.07.022 24011563 PMC3867811

[22] Kang Z Swaidani S Yin W Wang C Barlow JL Gulen MF. Epithelial cell-specific Act1 adaptor mediates interleukin-25-dependent helminth expulsion through expansion of Lin(-)c-Kit(+) innate cell population. Immunity. (2012) 36:821–33. 10.1016/j.immuni.2012.03.021 22608496 PMC3376903

[23] Swaidani S Bulek K Kang Z Liu C Lu Y Yin W. The critical role of epithelial-derived Act1 in IL-17- and IL-25-mediated pulmonary inflammation. J Immunol. (2009) 182:1631–40. 10.4049/jimmunol.182.3.1631 19155512 PMC3015148

[24] Xiao Y Jin J Chang M Nakaya M Hu H Zou Q. TPL2 mediates autoimmune inflammation through activation of the TAK1 axis of IL-17 signaling. J Exp Med. (2014) 211:1689–702. 10.1084/jem.20132640 24980047 PMC4113941

[25] Xiao Y Sun SC. TPL2 mediates IL-17R signaling in neuroinflammation. Oncotarget. (2015) 6:21789–90. 10.18632/oncotarget.4888 26318043 PMC4673121

[26] Yoneyama H Harada A Imai T Baba M Yoshie O Zhang Y. Pivotal role of TARC, a CC chemokine, in bacteria-induced fulminant hepatic failure in mice. J Clin Invest. (1998) 102:1933–41. 10.1172/JCI4619 9835618 PMC509145

[27] Pallett LJ Gill US Quaglia A Sinclair LV Jover-Cobos M Schurich A. Metabolic regulation of hepatitis B immunopathology by myeloid-derived suppressor cells. Nat Med. (2015) 21:591–600. 10.1038/nm.3856 25962123 PMC4458139

[28] Stravitz RT Kramer DJ. Management of acute liver failure. Nat Rev Gastroenterol Hepatol. (2009) 6:542–53. 10.1038/nrgastro.2009.127 19652652

[29] Ceci JD Patriotis CP Tsatsanis C Makris AM Kovatch R Swing DA. Tpl-2 is an oncogenic kinase that is activated by carboxy-terminal truncation. Genes Dev. (1997) 11:688–700. 10.1101/gad.11.6.688 9087424

[30] Patriotis C Makris A Bear SE Tsichlis PN. Tumor progression locus 2 (Tpl-2) encodes a protein kinase involved in the progression of rodent T-cell lymphomas and in T-cell activation. Proc Natl Acad Sci USA. (1993) 90:2251–5. 10.1073/pnas.90.6.2251 7681591 PMC46064

[31] Hedl M Abraham C. A TPL2 (MAP3K8) disease-risk polymorphism increases TPL2 expression thereby leading to increased pattern recognition receptor-initiated caspase-1 and caspase-8 activation, signalling and cytokine secretion. Gut. (2016) 65:1799–811. 10.1136/gutjnl-2014-308922 26215868 PMC5106344

[32] Chowdhury FZ Estrada LD Murray S Forman J Farrar JD. Pharmacological inhibition of TPL2/MAP3K8 blocks human cytotoxic T lymphocyte effector functions. PLoS ONE. (2014) 9:e92187. 10.1371/journal.pone.0092187 24642963 PMC3958505

[33] Watford WT Wang CC Tsatsanis C Mielke LA Eliopoulos AG Daskalakis C. Ablation of tumor progression locus 2 promotes a type 2 Th cell response in Ovalbumin-immunized mice. J Immunol. (2010) 184:105–13. 10.4049/jimmunol.0803730 19955521 PMC3044327

[34] Vougioukalaki M Kanellis DC Gkouskou K Eliopoulos AG. Tpl2 kinase signal transduction in inflammation and cancer. Cancer Lett. (2011) 304:80–9. 10.1016/j.canlet.2011.02.004 21377269

[35] Perugorria MJ Murphy LB Fullard N Chakraborty JB Vyrla D Wilson CL. Tumor progression locus 2/Cot is required for activation of extracellular regulated kinase in liver injury and toll-like receptor-induced TIMP-1 gene transcription in hepatic stellate cells in mice. Hepatology. (2013) 57:1238–49. 10.1002/hep.26108 23080298

[36] Li X Acuff NV Peeks AR Kirkland R Wyatt KD Nagy T. Tumor Progression Locus 2 (Tpl2) activates the Mammalian Target of Rapamycin (mTOR) pathway, inhibits Forkhead Box P3 (FoxP3) expression, and limits Regulatory T Cell (Treg) immunosuppressive functions. J Biol Chem. (2016) 291:16802–15. 10.1074/jbc.M116.718783 27261457 PMC4974392

[37] Acuff NV Li X Elmore J Rada B Watford WT. Tpl2 promotes neutrophil trafficking, oxidative burst, and bacterial killing. J Leukoc Biol. (2017) 101:1325–33. 10.1189/jlb.3A0316-146R 28356348 PMC5433858

[38] Wang YH Angkasekwinai P Lu N Voo KS Arima K Hanabuchi S. IL-25 augments type 2 immune responses by enhancing the expansion and functions of TSLP-DC-activated Th2 memory cells. J Exp Med. (2007) 204:1837–47. 10.1084/jem.20070406 17635955 PMC2118667

[39] Liu J Huang X Hao S Wang Y Liu M Xu J. Peli1 negatively regulates noncanonical NF-kappaB signaling to restrain systemic lupus erythematosus. Nat Commun. (2018) 9:1136. 10.1038/s41467-018-03530-3 29555915 PMC5859150

[40] Xiao Y Xu J Wang S Mao C Jin M Ning G. Genetic ablation of steroid receptor coactivator-3 promotes PPAR-beta-mediated alternative activation of microglia in experimental autoimmune encephalomyelitis. Glia. (2010) 58:932–42. 10.1002/glia.20975 20155818 PMC2903613

[41] Zhang X Wang Y Yuan J Li N Pei S Xu J. Macrophage/microglial Ezh2 facilitates autoimmune inflammation through inhibition of Socs3. J Exp Med. (2018) 215:1365–82. 10.1084/jem.20171417 29626115 PMC5940261

[42] Li D Cen J Chen X Conway EM Ji Y Hui L. Hepatic loss of survivin impairs postnatal liver development and promotes expansion of hepatic progenitor cells in mice. Hepatology. (2013) 58:2109–21. 10.1002/hep.26601 23813590

