# Supplementary material for: Tpl2 Protects Against Fulminant Hepatitis Through Mobilization of Myeloid-Derived Suppressor Cells
Source: Front Immunol. 2019 Aug 20;10:1980. doi: 10.3389/fimmu.2019.01980 (PMC6710335; doi:10.3389/fimmu.2019.01980)
Supplement: Supplementary file 1 [file Data_Sheet_1.PDF]

Supplemental Materials

Tpl2 protects against fulminant hepatitis through mobilization of myeloid-derived suppressor cells

by Xu *et al*.

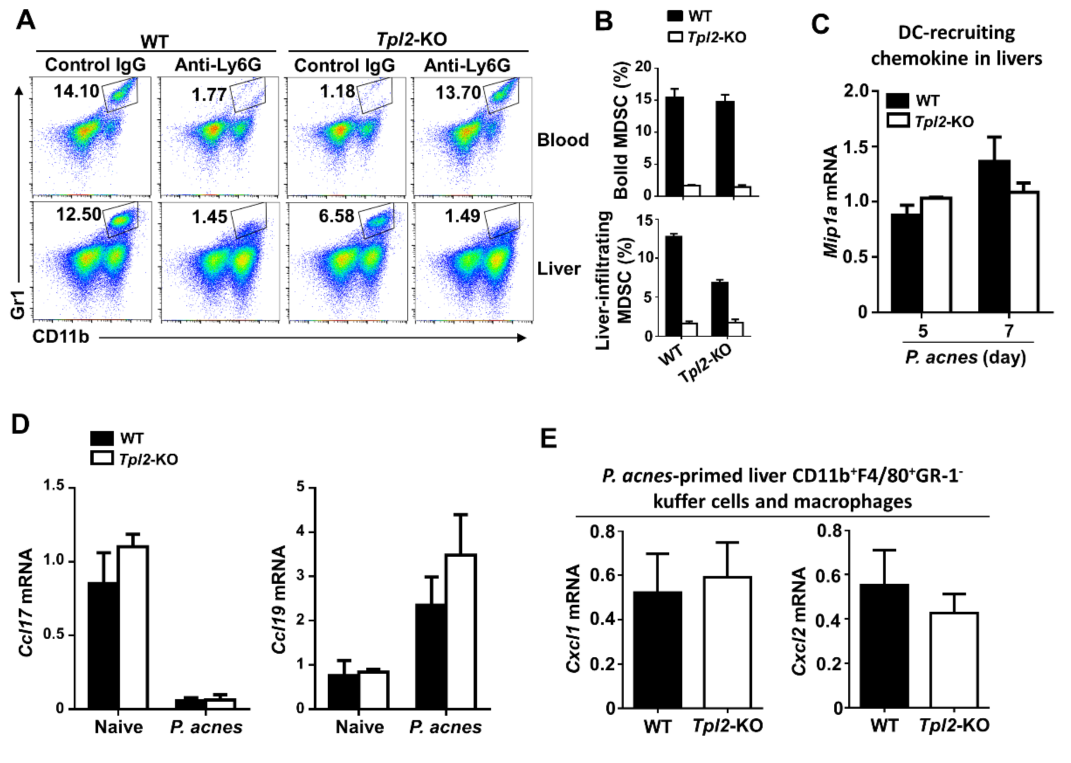

Supplementary Figure 1. MDSC depletion efficiency and chemokine expression in WT and Tpl2-deficient mice.

(**A, B**) Flow cytometry analysis of MDSC depletion efficiency in peripheral blood and livers of WT and Tpl2-deficient mice after i.v. injection of control and anti-Ly6G antibody. Data were presented as representative plots (**A**) and summary bar graphs (**B**). (**C-E**) QPCR analysis to determine the relative mRNA expression level of DC-recruiting chemokine *Mip1a* (**C**), or *Ccl17* and *Ccl19* (**D**) in livers of WT and *Tpl2*-KO that primed with or without *P. acnes* (n=4 mice/group), or *Cxcl1* and *Cxcl2* in *P. acnes*-primed WT and *Tpl2*-deficient liver CD11b^{+}F4/80^{+}Gr-1^{-} kuffer cells and macrophages (**E**). Data were normalized to a reference gene, *Actb*. Results are mean ± SD from three independent experiments.

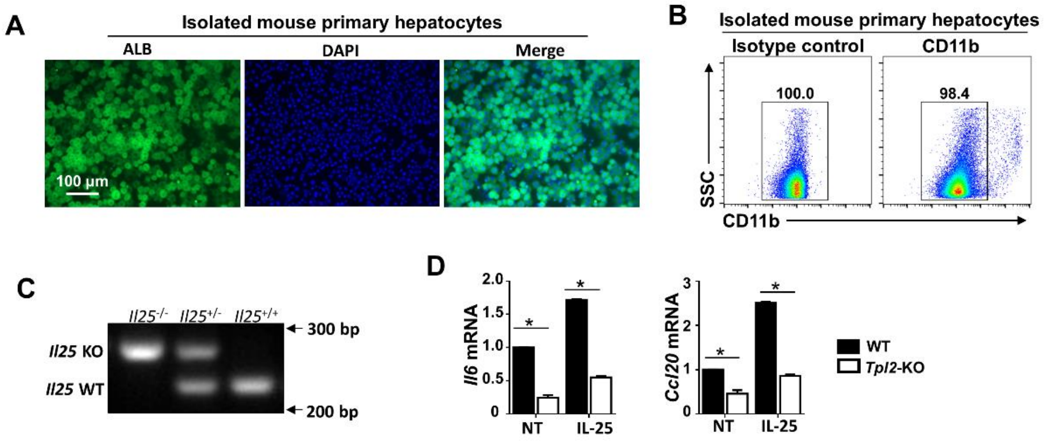

Supplementary Figure 2. Verification of isolated mouse primary hepatocytes and its response to IL-25 stimulation.

(**A**) Immunofluorescent analysis of the isolated mouse primary hepatocytes by staining with ALB. (**B**) Flow cytometry analysis of the isolated mouse primary hepatocytes by staining with CD11b. (**C**) Genotyping PCR identification of *Il25*$^{+/+}$, *Il25*$^{+/-}$ and *Il25*$^{-/-}$ mice. (**D**) QPCR analysis to determine the relative mRNA expression level of Il6 and Ccl20 in WT and Tpl2-deficient primary hepatocytes that left non-treated (NT) or stimulated with IL-25 for 8 h. Data were normalized to a reference gene, *Actb*. Results are mean ± SD from three independent experiments.

Supplementary Table 1. Primers used for real-time quantitative PCR

Genes	Forward primers (5'-3')	Reverse primers (5'-3')
m*Actb*	CGTGAAAAGATGACCCAGATCA	CACAGCCTGGATGGCTACGT
m*Ifng*	CAGCAACAGCAAGGCGAAA	CTGGACCTGTGGGTTGTTGAC
m*Tnf*	CATCTTCTCAAAATTCGAGTGACAA	CCAGCTGCTCCTCCACTTG
m*Cxcl1*	GCTGGCTTCTGACAACACT	CGCACAACACCCTTCTACT
m*Cxcl2*	CCCTGCCAAGGGTTGACTTC	GCAAACTTTTTGACCGCCCT
m*Ccl17*	TACCATGAGGTCACTTCAGATGC	GCACTCTCGGCCTACATTGG
m*Ccl19*	CCTGGGAACATCGTGAAAGC	TAGTGTGGTGAACACAACAGC
